# Supplementary material for: Intermediate-phase-assisted low-temperature formation of γ-CsPbI3 films for high-efficiency deep-red light-emitting devices
Source: Nat Commun. 2020 Sep 21;11:4736. doi: 10.1038/s41467-020-18380-1 (PMC7505955; doi:10.1038/s41467-020-18380-1)
Supplement: Supplementary file 1 — Supplementary Information [file 41467_2020_18380_MOESM1_ESM.pdf]

Supplementary Information

Intermediate-phase-assisted low-temperature formation of γ-$CsPbI_3$ films for high-efficiency deep-red light-emitting devices

Yi *et al*

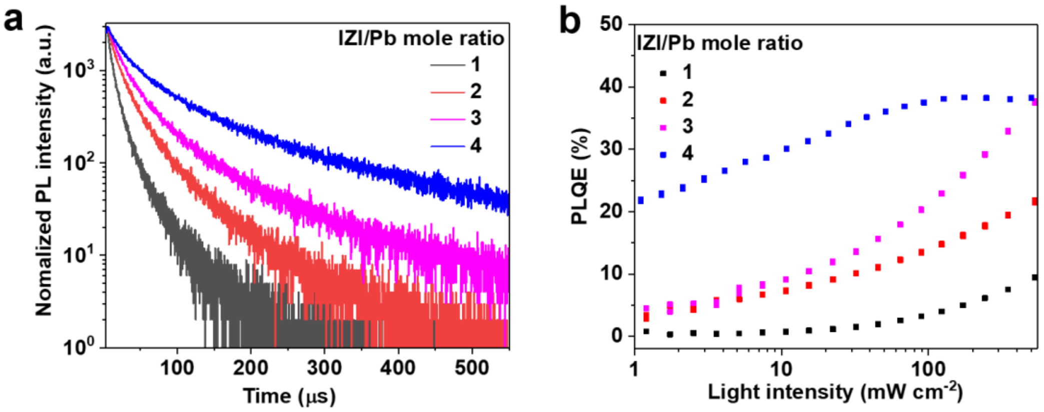

Supplementary Figure 1 Optical properties of $CsPbI_3$ films on ZnO/PEIE substrate**.** **(a)** Time-resolved PL for the $CsPbI_3$ films fabricated from precursor solutions with different mole ratio of IZI and PbI_2 (1:1, 2:1, 3:1, 4:1) under a fluence of 30 nJ cm^{-2}. (**b)** Excitation-intensity-dependent PLQE of $CsPbI_3$ films fabricated from precursor solutions with different mole ratio of IZI and PbI_2.

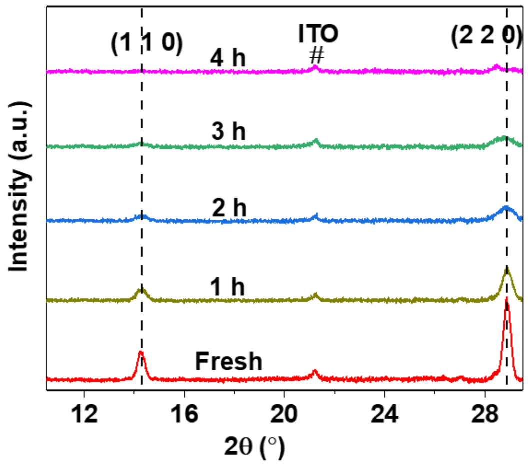

Supplementary Figure 2 XRD pattern of $CsPbI_3$ film without IZI after exposed in the air with 80 % relative humidity for various durations. The film is fabricated on ZnO/PEIE substrate at annealing temperature of 230 °C.

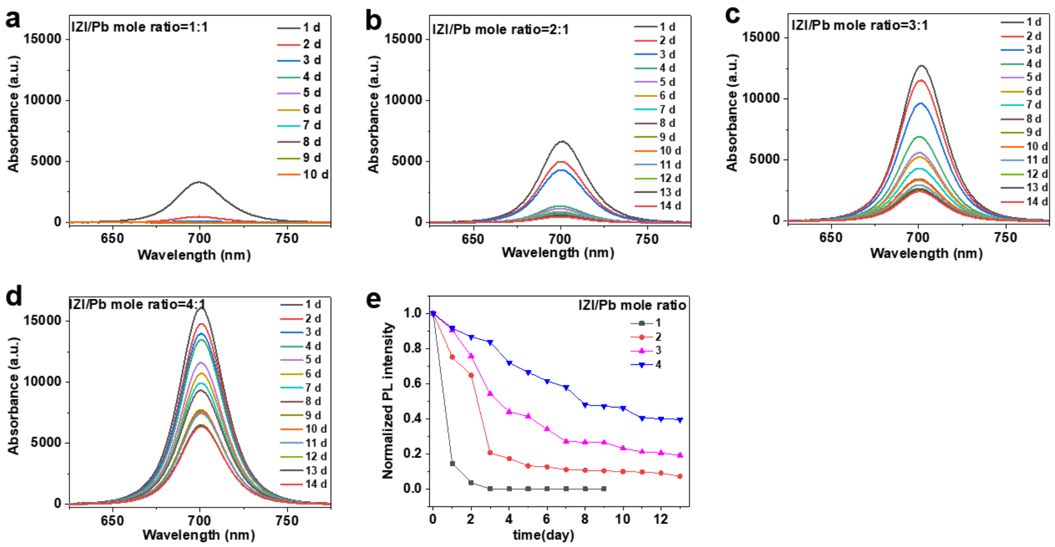

Supplementary Figure 3 PL spectrum of $CsPbI_3$ films fabricated from precursor solutions with different mole ratio of IZI and PbI_2 under 445 nm excitation with light intensity of 12.9 mW cm^{-2} in ambient air. (a) 1:1, (b)2:1, (c) 3:1, (d) 4:1. (e) Evolution of the normalized PL intensity of these films. Note that the PL intensity of $CsPbI_3$ films with mole ratio of 4 dropped to 50% over 8 days.

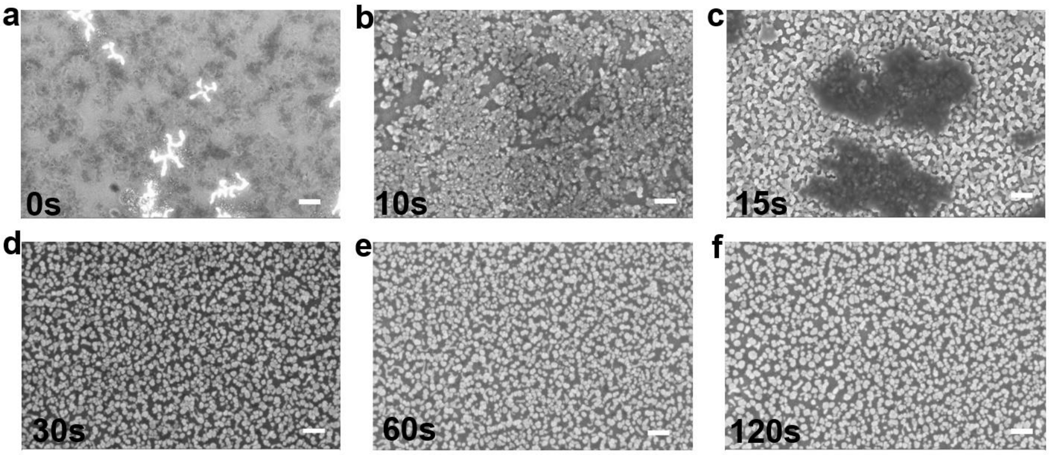

Supplementary Figure 4 SEM images of IZI-$CsPbI_3$ film annealed at 100 ºC for various time durations on ZnO/PEIE substrate. a) 0 s, b) 10 s, c) 15 s, d) 30 s, e) 60 s, f) 120 s. Scale bar, 400 nm.

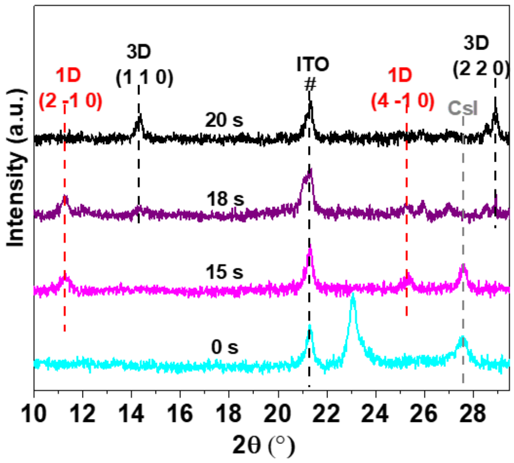

Supplementary Figure 5 (a) XRD patterns of IZI-$CsPbI_3$ film on ZnO substrate without the underneath ultrathin PEIE layer under various annealing time at 100 ºC.

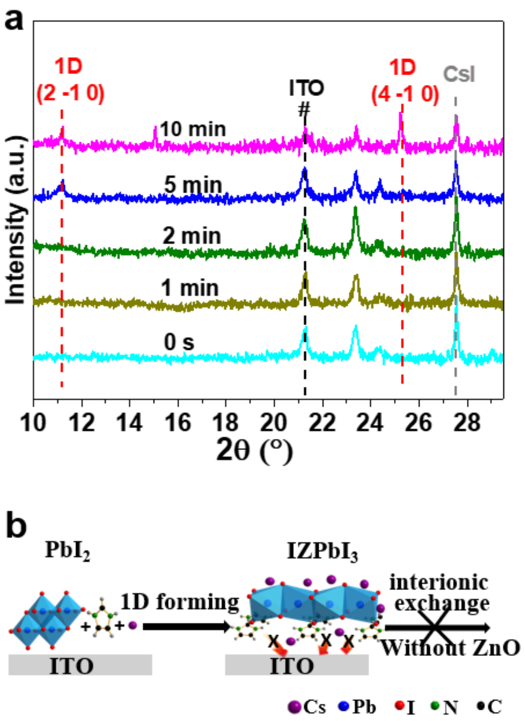

Supplementary Figure 6 (1) XRD patterns of IZI-$CsPbI_3$ film on ITO substrate with different annealing time at 100 °C. (b) Schematic diagram of the phase evolution of IZI-$CsPbI_3$ film on ITO substrate. Note that γ-$CsPbI_3$ cannot be formed without ZnO layer, and both the 1D phase (intermediate) and CsI remain unchanged.

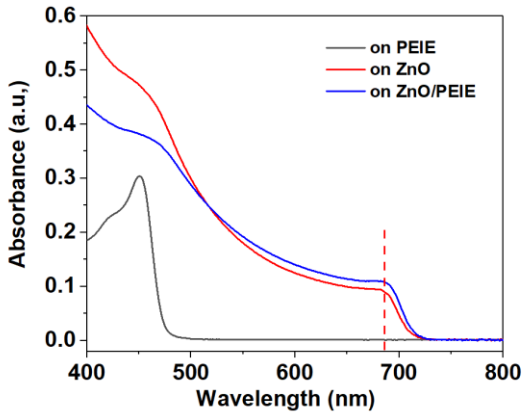

Supplementary Figure 7 (1) UV-vis spectrum of IZI-$CsPbI_3$ film on ITO/PEIE substrate (black line), ZnO substrate (red line) and ZnO /PEIE substrate (blue line), respectively. Note that γ-$CsPbI_3$ can be formed on ZnO/PEIE.

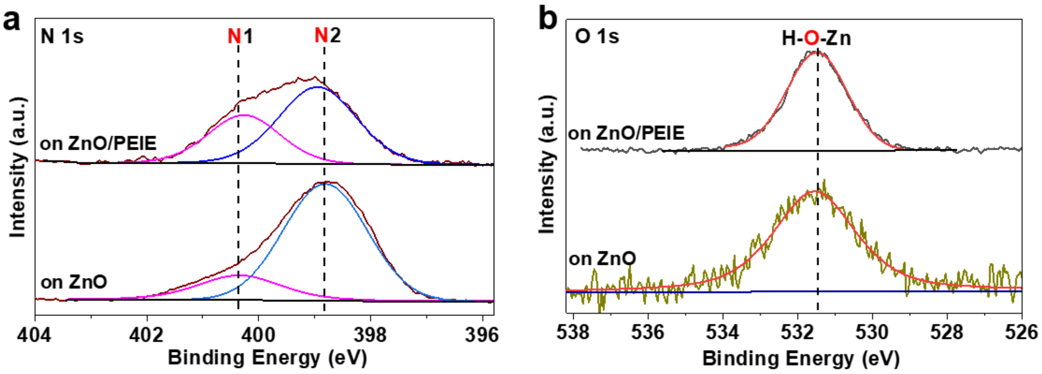

Supplementary Figure 8 XPS spectra of **(a)** N 1s and **(b)** O 1s for IZI-$CsPbI_3$ film on ZnO and ZnO/PEIE substrate, respectively. Note that modification PEIE does not prevent the deprotonation of the IZ^+ cation by ZnO.

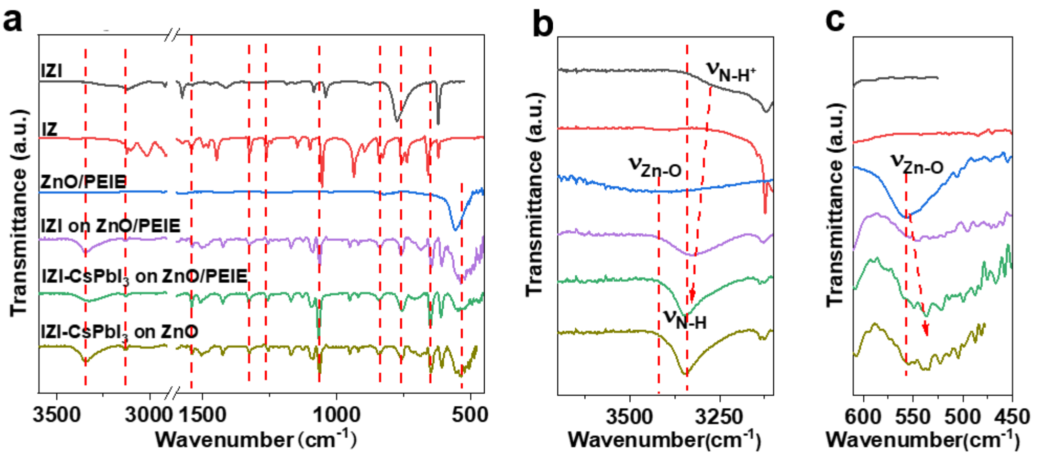

Supplementary Figure 9 ATR-FTIR spectra of IZI, IZ and IZI-$CsPbI_3$ films on ZnO/PEIE substrate (a), and magnified absorbance peak of the N-H stretching vibration (b) and Zn-O stretching vibration (c). The signature peak of imidazole (IZ) at about 3345 (ν_{N-H}), 3130 (ν_{C-H}), 1543 (δ_{NH}), 1328 (δ_{CH}), 1263 (ring breathing), 1055 (δ_{CH}), 841 (ring bend), 757 (γ_{CH}) and 658 (torsion) cm^{-1} are clearly observed. ν, stretching; δ, in-plane bending; γ, out-plane bending.

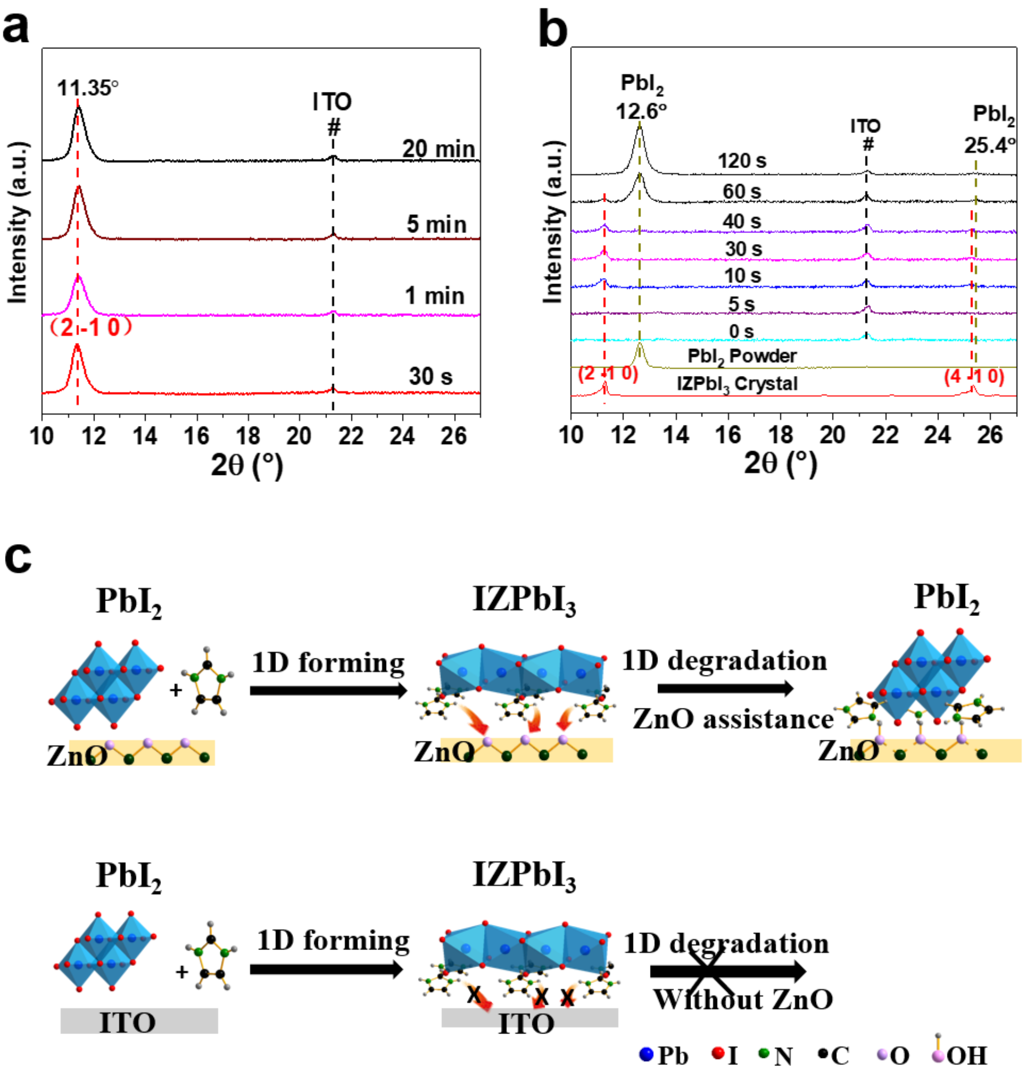

Supplementary Figure 10 XRD patterns of IZI-PbI_2 film on (a) ZnO /PEIE substrate and (b) ITO with different annealing time at 100 °C. (c) Schematic illustration of 1D perovskite formation and decomposition on different substrates. The intermediate phase $IZPbI_3$ was formed at early stage of thermal annealing process, and finally degraded to PbI_2 on the ZnO/PEIE substrate. On the contrary, the 1D phase retains unchanged even after 20 min thermal annealing on the ITO substrate.

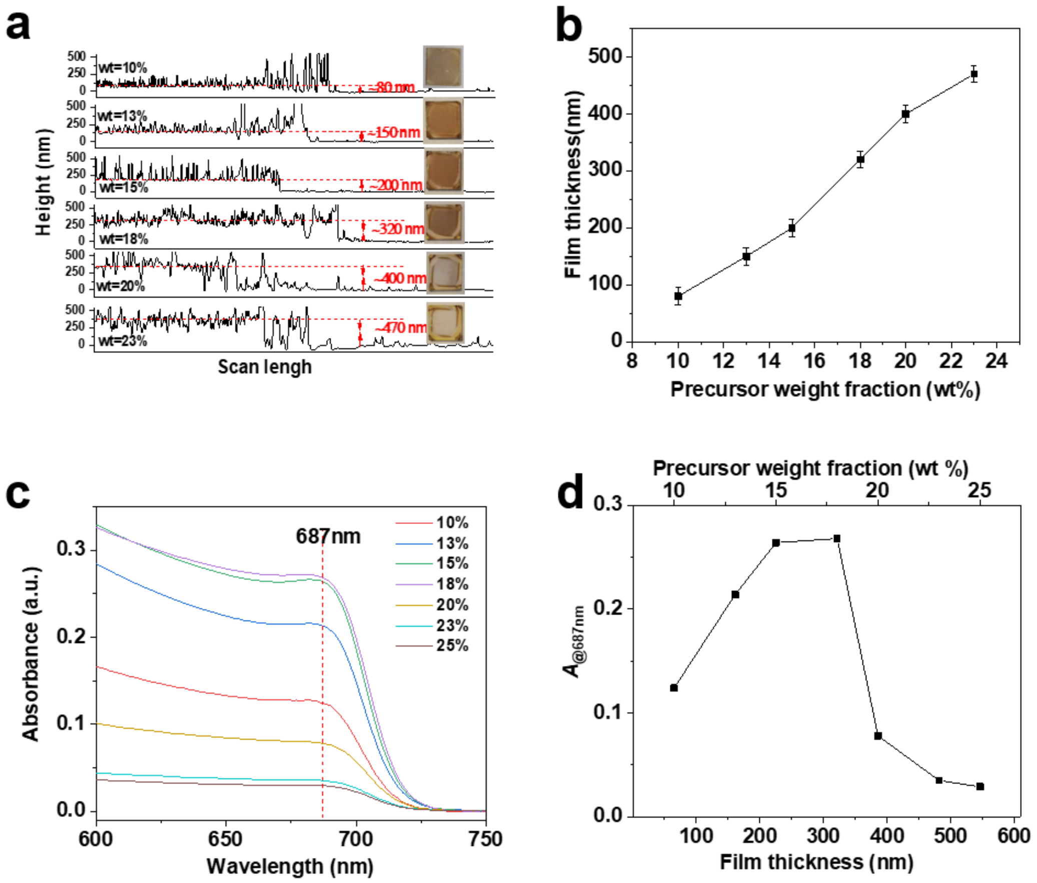

Supplementary Figure 11 Effectiveness of ZnO on the thickness of IZI-$CsPbI_3$ film fabricated with different weight fraction (wt %) of precursor solutions ranging from 10 % to 23 %. (a) thickness of IZI-$CsPbI_3$ film, as determined by profilometry. (b) IZI-$CsPbI_3$ film thickness as a function of the weight fraction (wt %) of precursor solutions. (c) UV-vis spectrum of IZI-$CsPbI_3$ film. (d) Relationship curve between the absorbance of $CsPbI_3$ black phase and film thickness.

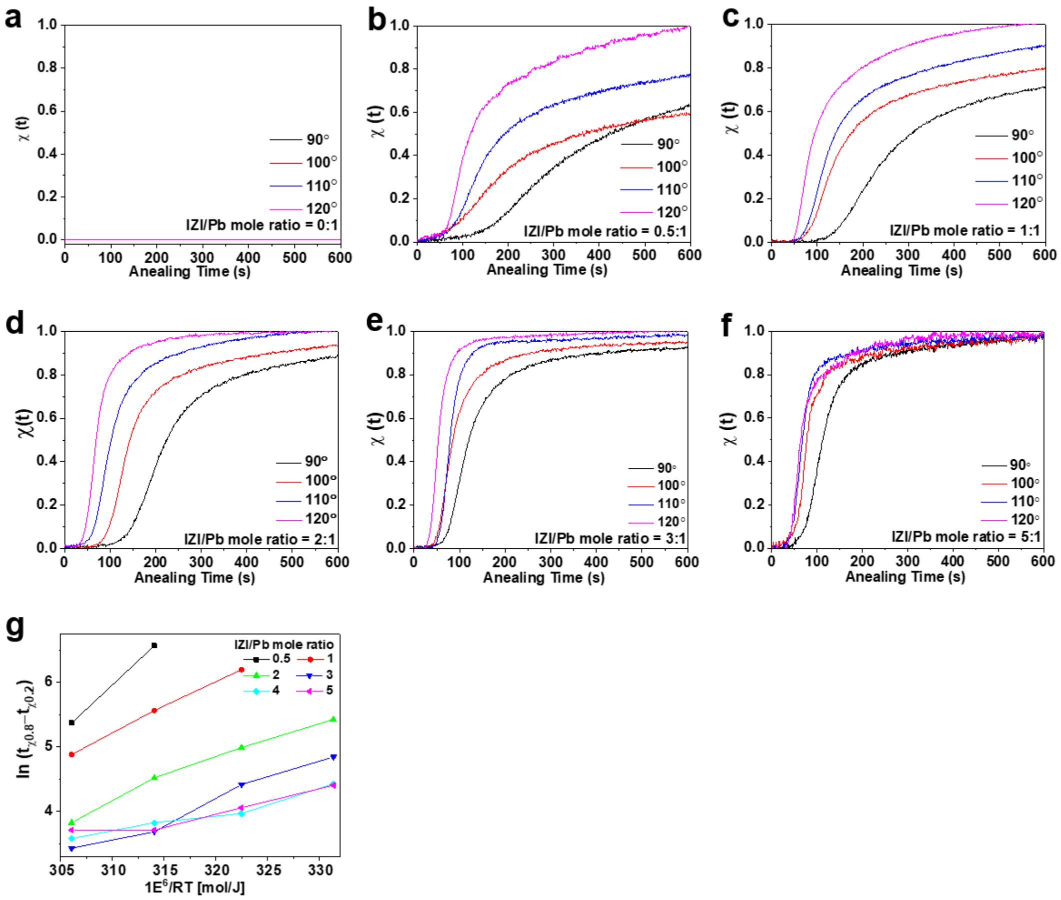

Supplementary Figure 12 Influence of annealing time on the γ-$CsPbI_3$ transition fraction $\chi(t)$ for films with mole ratio of (a) 0 : 1, (b) 0.5 : 1, (c) 1 : 1, (d) 2 : 1, (e) 3 : 1, (f) 5 : 1 at different temperature range from 90 °C to 120 °C. $\chi(t)$ is defined as $A(t)/A(t_{end})$, $A(t)$ represent the time-dependent absorbance at ~ 687 nm, $A(t_{end})$ represent the max absorbance. (g) Reaction kinetics fitted by Mittemeijer model for various IZI contents.

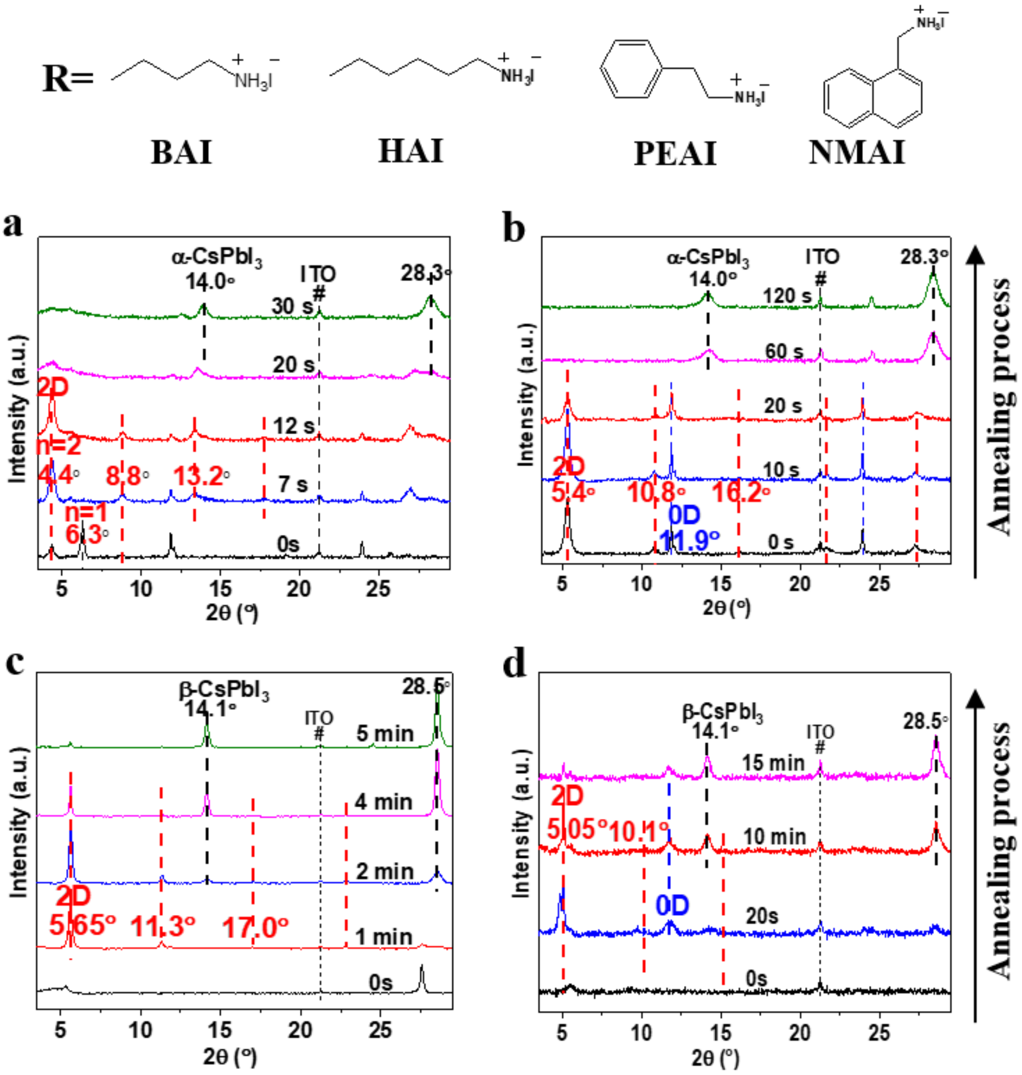

Supplementary Figure 13 XRD patterns of the intermediate two-dimensional (2D) perovskite (a) $(BA)_2PbI_4$, $(BA)_2CsPb_2I_7$, (b) $(HA)_2PbI_4$, (c) $(PEA)_2PbI_4$, (d) $(NMA)_2PbI_4$ during early stage of the thermal annealing process of RNH_3-$CsPbI_3$ films on ZnO substrate at 100 °C. The corresponding XRD peak of intermediate at 6.3°, 4.4°, 5.4°, 5.65°, 5.05°, respectively. Upon further annealing, both the XRD peaks of intermediate phase disappear, and XRD peaks (14.0° / 14.1° and 28.3° / 28.5°) of $CsPbI_3$ black phase appear. 0D: perovskite Cs_4PbI_6.

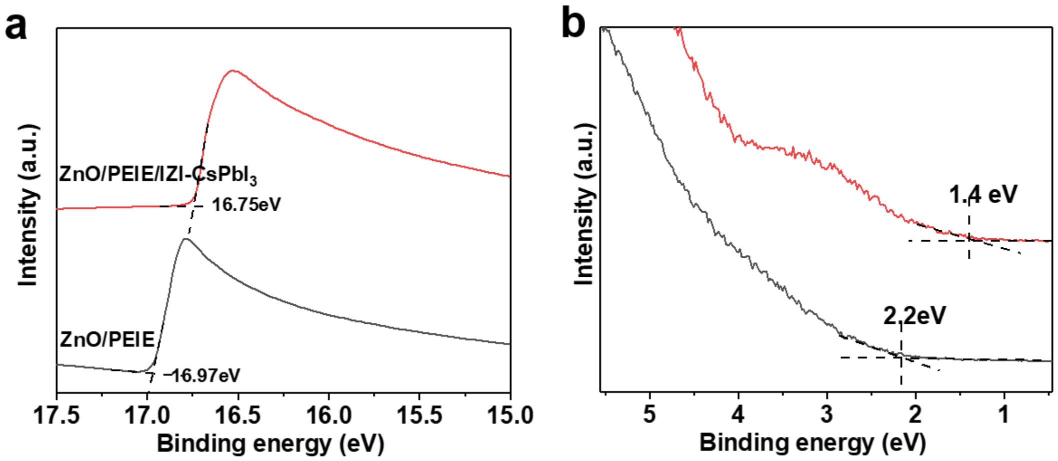

Supplementary Figure 14 UPS spectra of ZnO/PEIE substrate and IZI-$CsPbI_3$ film spin-coated on ZnO/PEIE substrate.

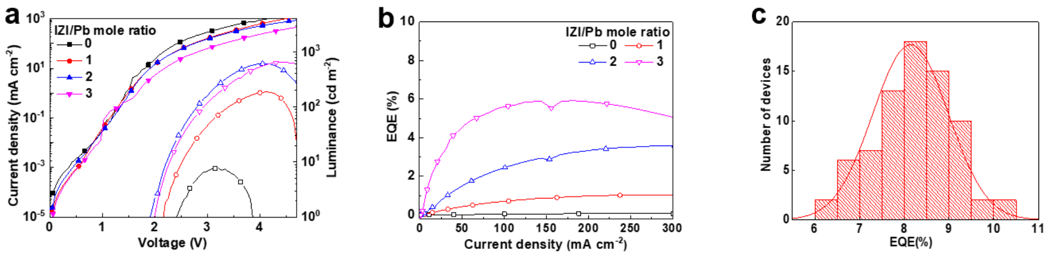

Supplementary Figure 15 Optoelectronic characteristics of $CsPbI_3$ LEDs based on different mole ratio of IZI and PbI_2 (0 : 1, 1 : 1, 2 : 1, 3 : 1). (a) Current density and luminance versus voltage (*J*-*L*-*V*). (b) EQE versus current density (*EQE*-*J*) curves. (c) Histogram of peak EQE measured from 75 device with IZI/Pb mole ratio of 4 : 1, which shows an average of EQE of 8.1 % with a relative standard deviation (RSD) of 10.4 %.

Supplementary Table 1. Crystallographic Data for intermediate phase ($IZPbI_3$)

Temperature (K)	150
CCDC	1972776
Empirical Data	$C_6H_{10}N_4Pb_2I_6$
Formula Weight	1314.00
Crystal system	hexagonal
Space group	*P*-62m (No. 189)
a (Å)	15.3821(4)
b (Å)	15.3821(4)
c (Å)	7.9832(5)
α=β (°)	90
γ (°)	120
V (Å^3)	1635.83(15)
Dcalcd (g/cm^3)	4.035
Z value	1
Temperature (K)	150
μ (mm^{-1})	23.907
F(000)	1677.0
R_1/wR_2 [I>2σ(I)]	0.0305/0.0781
R indices (all data)	0.0355
Goodness-of-fit on F^2	1.084
